# The Hospital Conference at Newcastle: Some Lessons and Suggestions

**Published:** 1914-06-27

**Authors:** 


### THE HOSPITAL CONFERENCE AT NEWCASTLE.

### Some Lessons and Practical Suggestions.

The arrangements for this year's meeting of the British Hospitals Association Conference at Newcastle-on-Tyne reflect the greatest credit upon those who were responsible for them. No better debating hall could be found for a meeting of the kind than that afforded by the most attractive and spacious library attached to the Royal Infirmary. This library is light, airy, and well ventilated, its acoustic properties were of the best, and everything contributed to promote the comfort of speakers and audiences alike.

The Lord Mayor showed generous hospitality and a cordial and powerful interest in the proceedings of the meeting. The committee of the infirmary gave an excellent luncheon to those who attended the meeting, and Dr. Hume's luncheon party on the following day, which was attended by the Lord Mayor and Sheriff, was greatly enjoyed by everyone present. Mr. Roden Orde, the able superintendent and secretary of the Newcastle Royal Infirmary, worked hard and successfully to make the proceedings a success, and to him are due the grateful thanks and gratitude of the members of the British Hospitals Association and of the City of Newcastle, for they passed off without a hitch, and so added gratifying testimony to the generous hospitality and organising power which has long characterised the city of Newcastle-on-Tyne and its inhabitants. The honorary secretaries of the Association showed commendable energy and zeal, which proves them to be the right men in the right place.

### The Meetings and Speakers.

We have received from a correspondent an article conveying his impressions of the debates. We have not room for it this week, but we may usefully utilise some of the points which he makes. His first point is that in the earlier days of the Annual Conferences of this Association there was a tendency for the same speakers to attend, when a handful of them, whose interests were mostly confined to the smaller institutions, were apt to repeat themselves year by year. At Newcastle this feature was entirely absent, and the entire proceedings were freed from any evidence of desire on the part of anyone present to advertise either themselves or their hobbies. This is matter for congratulation, for it is of the first importance that the Council shall so arrange the business as to afford the maximum guarantees to those who attend and are anxious to hear the informative experience of hospital veterans like Mr. Howard Collins and Mr. Carnt, and many others who might be mentioned. The best men could then rest assured that the practical side of hospital work and administration would be properly brought out in the course of the subjects debated.

In the early days of the Association it was the practice to divide the papers into sections, so that each topic might be dealt with from every point of view. It was part of the plan to select a chairman to preside at each section who had an intimate knowledge of the subjects to be dealt with in the papers, and who was thus able to direct the discussions and to sum up the result in a short practical speech at the close of each debate. We think, seeing that the educational side of the British Hospitals Association should be increasingly provided for, that the Council will be well advised to consider whether they cannot revert to some such plan at future conferences of the Association.

One difficulty which has to be met is to arrange a time limit or some scheme by which speakers shall be brought to condense what they have to say into as few words as possible. One step towards the attainment of this end would be to arrange, when readers of papers were selected, that the MS. of these papers should be in the hands of the honorary secretaries at least three weeks before the date fixed for the annual meeting. This would enable the honorary secretaries to get the papers into type, and facilitate the adoption of another plan of equal practical value to that of the choice of subjects for the chief papers contributed. This is to arrange for the debate upon the important papers to be opened by two or three selected speakers who are knowledgeable and capable of dealing with the points raised in a clear, practical manner. Each selected speaker should then have a copy of the paper to be debated sent to him at least a fortnight before the date when it is to be read. Anyone who reads Dr. Hume's paper, for example, will appreciate the importance of this suggestion. Proofs of this paper only came to hand on the morning when it was to be read, and it was quite impossible for anyone, in the short space of one hour, to study it carefully and to debate it effectively. The result was that the main speeches were longer than they would otherwise have been.

There were several institutions to see in Newcastle, including the Royal Infirmary, the Children's Hospital, and the new hospital wards of the Newcastle Workhouse. For some reason the afternoon of the second day was not given up to the new hospital wards at the workhouse. We hope to publish a full account of these wards and the lessons they teach, but we may here express regret that an opportunity to visit them should have been delayed until Saturday morning, when most of the members present at the meeting had arranged to leave Newcastle. The chairman of the Board of Guardians was in attendance, with the principal medical officer, to receive members of the Association, and did everything possible to enable those who visited the new wards to see and compare them with the old buildings where sick patients and surgical cases are still under treatment, though the latter ought to be moved out of them without a moment's avoidable delay. We congratulate the chairman and the Board of Guardians upon their public spirit in submitting their new wards to inspection, and feel sure that the results of their courtesy and courage will bear gratifying fruit.

